# Frontal White Matter Hyperintensities and Executive Functioning Performance in Older Adults

**DOI:** 10.3389/fnagi.2021.672535

**Published:** 2021-06-28

**Authors:** Emanuel M. Boutzoukas, Andrew O'Shea, Alejandro Albizu, Nicole D. Evangelista, Hanna K. Hausman, Jessica N. Kraft, Emily J. Van Etten, Pradyumna K. Bharadwaj, Samantha G. Smith, Hyun Song, Eric C. Porges, Alex Hishaw, Steven T. DeKosky, Samuel S. Wu, Michael Marsiske, Gene E. Alexander, Ronald Cohen, Adam J. Woods

**Affiliations:** ^1^Center for Cognitive Aging and Memory, McKnight Brain Institute, University of Florida, Gainesville, FL, United States; ^2^Department of Clinical and Health Psychology, College of Public Health and Health Professions, University of Florida, Gainesville, FL, United States; ^3^Department of Neuroscience, College of Medicine, University of Florida, Gainesville, FL, United States; ^4^Department of Psychology and Evelyn F. McKnight Brain Institute, University of Arizona, Tucson, AZ, United States; ^5^Department Psychiatry, College of Medicine, University of Arizona, Tucson, AZ, United States; ^6^Department of Neurology, College of Medicine, University of Arizona, Tucson, AZ, United States; ^7^Department of Neurology, College of Medicine, University of Florida, Gainesville, FL, United States; ^8^Department of Biostatistics, College of Public Health and Health Professions, College of Medicine, University of Florida, Gainesville, FL, United States; ^9^Department of Psychiatry, Neuroscience and Physiological Sciences Graduate Interdisciplinary Programs, and BIO5 Institute, University of Arizona and Arizona Alzheimer's Disease Consortium, Tucson, AZ, United States

**Keywords:** cognitive aging, executive function, NIH toolbox, white matter hyperintensities, region-specific hyperintensities, frontal lobes, FLAIR MRI

## Abstract

Frontal lobe structures decline faster than most other brain regions in older adults. Age-related change in the frontal lobe is associated with poorer executive function (e.g., working memory, switching/set-shifting, and inhibitory control). The effects and presence of frontal lobe white matter hyperintensities (WMH) on executive function in normal aging is relatively unknown. The current study assessed relationships between region-specific frontal WMH load and cognitive performance in healthy older adults using three executive function tasks from the NIH Toolbox (NIHTB) Cognition Battery. A cohort of 279 healthy older adults ages 65–88 completed NIHTB and 3T T1-weighted and FLAIR MRI. Lesion Segmentation Toolbox quantified WMH volume and generated lesion probability maps. Individual lesion maps were registered to the Desikan-Killiany atlas in FreeSurfer 6.0 to define regions of interest (ROI). Independent linear regressions assessed relationships between executive function performance and region-specific WMH in frontal lobe ROIs. All models included age, sex, education, estimated total intracranial volume, multi-site scanner differences, and cardiovascular disease risk using Framingham criteria as covariates. Poorer set-shifting performance was associated with greater WMH load in three frontal ROIs including bilateral superior frontal (left β = −0.18, FDR-*p* = 0.02; right β = −0.20, FDR-*p* = 0.01) and right medial orbitofrontal (β = −0.17, FDR-*p* = 0.02). Poorer inhibitory performance associated with higher WMH load in one frontal ROI, the right superior frontal (right β = −0.21, FDR-*p* = 0.01). There were no significant associations between working memory and WMH in frontal ROIs. Our study demonstrates that location and pattern of frontal WMH may be important to assess when examining age-related differences in cognitive functions involving switching/set-shifting and inhibition. On the other hand, working memory performance was not related to presence of frontal WMH in this sample. These data suggest that WMH may contribute selectively to age-related declines in executive function. Findings emerged beyond predictors known to be associated with WMH presence, including age and cardiovascular disease risk. The spread of WMH within the frontal lobes may play a key role in the neuropsychological profile of cognitive aging. Further research should explore whether early intervention on modifiable vascular factors or cognitive interventions targeted for executive abilities may help mitigate the effect of frontal WMH on executive function.

## Introduction

Older adults are at risk for decline in executive function. Executive functions are skills essential for everyday tasks including evaluating and manipulating mental information, regulating thoughts and emotions, and making decisions in a goal-directed manner (Lezak et al., 2004). The aging process involves a variety of neuroanatomical changes which may be related to cognitive changes. Micro-and-macrostructural damage to white matter may contribute to functional decline in older adults because of its integral role in neurotransmission (Liu et al., 2017). White matter hyperintensities (WMH) are a common age-related macrostructural brain change shown on T2-wieghted magnetic resonance imaging (MRI) which represent localized white matter damage. Previous studies demonstrated that WMH occur in normal aging, increase with age, and represent an accumulation of small-vessel cerebrovascular disease (Brickman et al., 2014). The presence and extent of WMH are important contributors to adverse clinical outcomes by lowering the threshold for the clinical expression of dementia (Alber et al., 2019). Thus, the examination of WMH's effect on normal age-related cognitive decline, specifically executive function, is an emerging area of research.

Studies assessing relationships between whole-brain increases in WMH and declines in cognition have most commonly examined executive function, memory, and processing speed domains (Bolandzadeh et al., 2012; Brugulat-Serrat et al., 2020). More recent research suggests regional WMH may be a sensitive metric for identifying differential declines across cognitive domains (Prins and Scheltens, 2015). However, only a small number of studies have assessed associations between regional WMH and cognitive function in healthy individuals (Desmond, 2002; Bolandzadeh et al., 2012; Lampe et al., 2017; Brugulat-Serrat et al., 2020). Voxel-wise analyses have demonstrated relationships between increased periventricular and deep white matter hyperintensities and cognitive decline in healthy individuals and age-related diseases, likely due to disruption of long association white matter tracts (Wakefield et al., 2010; Smith et al., 2011; Lockhart et al., 2012; Birdsill et al., 2014; Dhamoon et al., 2018). More recent research suggests that, in healthy individuals, superficial WMH are more strongly associated with performance in cognitive domains, including memory and executive functioning, while the presence of periventricular/deep WMH might be mediated by age or other risk factors for WMH (Brugulat-Serrat et al., 2020). Therefore, the impact of the spread and extent of WMH from periventricular to superficial white mater regions requires further exploration (Bolandzadeh et al., 2012).

Executive functions are one domain that encompass several cognitive processes, yet studies often examine this domain as a singular construct, which may impede our understanding of these unique subcomponents. The subcomponents are commonly described as three latent constructs: set-shifting (task switching), inhibition (suppressing prepotent responses), and information updating (working memory; Miyake et al., 2000). The primary neural correlate of executive functions are the frontal lobes. In aging, the frontal lobes are disproportionately vulnerable to structural and functional changes. Age-related changes in executive function have been attributed to disruptions in frontal lobe structural integrity, and preliminary research suggests that subcomponents of executive function have distinctive neuroanatomical substrates (Collette et al., 2005; Kennedy and Raz, 2009). Yet, it is still unclear how patterns of frontal WMH are related to declines in subcomponents of executive functions in normal aging. Characterizing the relationship between the presence of both superficial and periventricular/deep region-specific WMH, cardiovascular disease (CVD) risk, and executive function decline prior to the onset of pathological aging may provide a critical biomarker for identifying risk of transition from normal to pathological aging.

A recent systematic review of WMH studies examining its relationship with cognition found that studies have most used single-domain traditional neuropsychological measures to examine executive function. In their examinations, the most common tests were traditional paper-pencil evaluations including the Trail-Making Test, Stroop Test, and Verbal Fluency Test (Bolandzadeh et al., 2012). The National Institutes of Health Toolbox (NIHTB) Cognition Battery provides a well-validated comprehensive computerized battery that uses item response theory to efficiently assess several cognitive domains (Weintraub et al., 2013). Additionally, it provides multifaceted data not readily obtainable *via* traditional neuropsychological testing, including reaction time and response accuracy (Heaton et al., 2014). The NIHTB provides a cohesive battery for assessing three key subcomponents of executive function, which are yet to be explored in the context of WMH and age-related executive function declines. Deficits to any of the three subcomponents has the potential to interfere with performing daily activities and maintaining functional independence, as they limit the ability to quickly process or transform mental information (Murman, 2015).

The present study set out to examine contributions of region-specific frontal WMH load on NIHTB executive function subtest performance as a potential biomarker for risk of increased cognitive decline. We restricted our analyses to the frontal lobe because of its disproportional impact in the aging process (Raz, 2000; Raz et al., 2005), and prior findings in functional studies demonstrating distinct neuroanatomical correlates of executive functions (Collette et al., 2005). Frontal WMH were quantified using an atlas-based approach to parse out distinct frontal regions. We hypothesized a relationship between poorer switching/set-shifting performance and higher WMH in left middle frontal, left inferior frontal, and periventricular/deep frontal regions, as measured by the Dimensional Change Card Sorting Test (DCCS). We hypothesized that poorer performance in inhibitory control would be associated with higher WMH in left middle frontal, bilateral inferior frontal, and periventricular/deep frontal regions, as measured by the Flanker Inhibitory Control and Attention Test (Flanker). We hypothesized that poorer working memory performance would be associated with higher WMH in bilateral middle frontal, superior frontal, inferior frontal, and periventricular/deep frontal regions, as measured by the List Sorting Working Memory Test (List Sorting). We assessed relationships beyond contributions of age, sex, years of education, differences in MRI scanner, head size, and presence of cardiovascular disease risk.

## Materials and Methods

### Participants

Two hundred and ninety healthy older adults ranging from 65 to 88 years old (mean age = 71.5 ± 5.1, 181 females; mean education = 16.3 ± 2.4) took part in the current study. Participants were recruited at the University of Florida (*n* = 191) and at the University of Arizona (*n* = 99) for the Stimulated Brain (K01AG050707) and the Augmenting Cognitive Training in Older Adults (ACT, R01AG054077) studies (Woods et al., 2018). Participants were recruited *via* newspaper, direct mail, radio and television advertisements, local community outreach events, and through research contact registries. Inclusion and exclusion criteria for both studies were identical. The exclusionary criteria for these protocols limited the sample to right handers with no contra-indications for MRI, no history of major psychiatric illness, no history of brain or head injury resulting in loss of consciousness >20 min, and no formal diagnosis or evidence of Mild Cognitive Impairment (MCI), dementia, or neurological brain disease. The study was approved by and performed according to the policies of the Institutional Review Boards at the University of Florida (UF) and the University of Arizona (UA). Participants engaged in the informed consent process prior to the initiation of study activities. All data in the current study were acquired from the baseline visit of the involved studies. At their baseline visit, participants completed a battery of cognitive assessments, medical history and mood questionnaires, and a multimodal MRI scan. Eleven subjects were excluded from analysis due to use of different MRI head coil (20-Channel), or outlier data (>3 standard deviations from the mean) on cognitive measures (*n* = 6). Analyses included a total of 279 participants (see Table 1 for sample demographics).

**Table 1:** Participant demographics.

**Demographics**	**Mean/SD**
Age	71.6 ± 5.0
Education (number of years)	16.46 ± 2.4
**Sex**	***N*** **(%)**
Males	104 (37.3%)
Females	175 (62.7%)
**Race**	***N*** **(%)**
White	247 (88.5%)
African American or Black	11 (3.9%)
American Indian/Alaska Native	7 (2.5%)
Asian	2 (0.7%)
Native Hawaiian or Other Pacific Islander	1 (0.4%)
More than one race	5 (1.8%)
Did not disclose	6 (2.2%)
**Ethnicity**	***N*** **(%)**
Hispanic or Latino	21 (7.5)
Not Hispanic Latino	257 (92.1)
Did not disclose	1 (0.4)

### National Institutes of Health Toolbox

All participants received the NIHTB Cognition Battery as an assessment of multiple cognitive domains. We obtained unadjusted scaled scores for the respective executive function subtests. A brief description of these tasks follows, which are documented in detail elsewhere (Heaton et al., 2014).

#### Executive Function Measures

The NIHTB Cognition Battery includes three subtests measuring aspects of executive function (set-shifting, inhibition, and working memory). The Dimensional Change Card Sort Test (DCCS) assesses switching and set-shifting ability. In this task, a target stimulus displays while two different stimuli appear side-by-side at the bottom of the screen. The participant must match one of the two new stimuli to the target stimulus based on the rule presented, which switches between trials to match on either shape or color. The Flanker Inhibitory Control and Attention Test (Flanker) measures executive inhibition of pre-potent responses. In this task, participants must inhibit automatic responses and select the direction of a central stimulus (an arrow) flanked by similar stimuli on either side facing the same or different directions. The List Sorting Working Memory Test (List Sorting) requires participants to temporarily store and manipulate information. Participants must order a sequence of visual and auditory stimuli by size (smallest to largest) and category (foods and animals). The list increases in complexity, as items presented increases. Additionally, participants must sort the items by size by one category (either foods or animals presented in a single trial), and then by size in two categories (foods and animals presented in a single trial).

#### Outlier Analysis

The NIHTB executive function subtest scores were transformed to z-statistic to identify outliers. Six participants were removed from final analysis: Four outliers on the Flanker task (*z*-statistic = −3.0, −3.2, −3.2, −4.5), one outlier on the List Sorting working memory task (*z*-statistic = 3.3), and one outlier on both the Flanker and DCCS task (Flanker *z*-statistic = −4.6, DCCS *z*-statistic = −3.5). Mean performance for each site is located in Table 2.

**Table 2:** NIH toolbox scores by site.

**NIH toolbox**	**Site**	***N***	**Mean/SD**
Dimensional change card sorting	UF UA	180 99	100.8 ± 6.6 101.3 ± 6.7
Flanker inhibitory attention	UF UA	180 99	94.0 ± 5.5 96.2 ± 4.7
List sorting working memory	UF UA	180 99	98.8 ± 8.6 99.6 ± 8.3

*NIH Toolbox scores are unadjusted standard scores*.

### MRI Procedures

#### MRI Acquisition

All MRI was collected using either a 3-Tesla Siemens Magnetom Prisma with a 64-channel head coil or 3-Tesla Siemens Magnetom Skyra scanner with a 32-channel head coil at UF and UA, respectively. Each study site used identical sequence parameters and followed the same scanning protocol. Foam padding around the head aimed to limit motion during the scan. Earplugs minimized noise while inside the scanner. High resolution T1-weighted 3D Magnetization Prepared Rapid Gradient Echo (MPRAGE; repetition time [TR] = 1,800 ms; echo time [TE] = 2.26 ms; 1.0 × 1.0 × 1.0 mm^3^ voxels; 176 slices; field of view [FOV] = 256 × 256 mm; flip angle [FA] = 8; time = 3 min and 3 s) and T2-weighted Fluid Attenuated Inversion Recovery (FLAIR; TR = 7,000 ms; TE = 101 ms; 1.0 × 1.0 × 2.5 mm^3^ voxels; 55 slices; FOV = 256 × 256 mm; FA = 120; time = 3 min and 9 s) were collected as part of the MRI session.

#### MRI Processing

**Figure 1:**
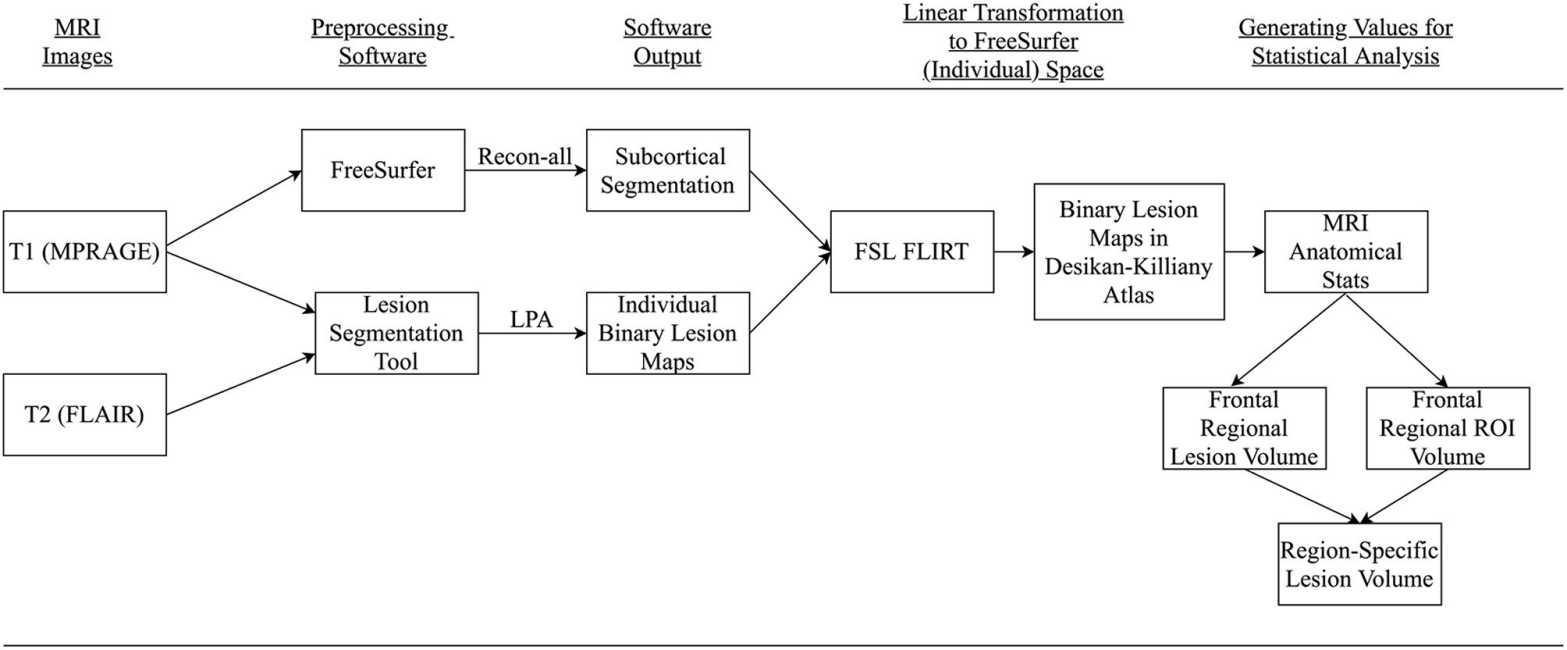
Summary conceptual model of MRI processing. Participant's MPRAGE and FLAIR were collected and processed using FreeSurfer and Lesion Segmentation Toolbox. Resultant images were transformed to FreeSurfer space, the Desikan-Killiany atlas, *via* FSL FLIRT. Regional-specific lesion volumes were calculated for final analysis.

The FLAIR and MPRAGE series were processed using two freely available software applications, the Lesion Segmentation Tool (LST) for SPM12 (www.statistical-modeling.de/lst.html) and the FreeSurfer 6.0 imaging analysis suite (http://surfer.nmr.mgh.harvard.edu/). Figure 1 provides an overview of our processing pipeline. Although only the Lesion Prediction Algorithm only requires the FLAIR, the optional MPRAGE was input for improved segmentation (LPA; Schmidt et al., 2012, Chapter 6.1). Previous research has demonstrated the accuracy of the LPA as an automated processing method for detecting WMH in healthy and neurodegenerative disease populations (de Sitter et al., 2017; Egger et al., 2017; Ribaldi et al., 2021). Technical details of LST's procedures for lesion identification are detailed in their documentation (https://statistical-modelling.de/LST_documentation.pdf). Briefly, LPA segments brain tissue (i.e., gray matter, white matter, and cerebrospinal fluid) on the MPRAGE. It registers the FLAIR to MRPAGE and calculates lesion maps. In addition to implementing a similar lesion belief map to the Lesion Growth Algorithm (LGA) (Schmidt et al., 2012), the LPA uses a spatial covariate that accounts for voxel specific changes in lesion probability. This technique calculates an estimate for the lesion probability at each voxel, which captures relationships between neighboring voxels. After processing, LST-generated binary lesion maps were exported for use with other software. Consistent with previous research a lesion threshold of 0.30 created conservative binary lesion maps (Birdsill et al., 2014).

For quality assurance, total lesion volume and region-specific results were generated for both LGA and LPA. The LPA was used based on visual comparison between the two lesion algorithms (Supplementary Figure 1). Values generated from the pipelines were highly correlated (*r* = 0.98) and regional findings were consistent. We visually inspected individual lesion segmentation results for accuracy. Lesion Segmentation Tool provides side-by-side “overlay” images for comparison of original T2-image with lesion segmentation results. These images can be viewed in an HTML report and are supplied slice-by-slice in PNG format for each participant. To ease visual inspection, the study team (EB & AA) merged each individual subjects' LST-generated overlay images into video (.gif) format, at a speed of 3 slices/second. All participants' lesion segmentation results were visually reviewed, and no participants were removed for poor lesion classification.

FreeSurfer's default processing stream uses the MPRAGE as its primary input. The technical details of FreeSurfer's procedures for morphometric analysis are detailed in prior publications (Dale et al., 1999; Fischl et al., 1999, 2004; Fischl and Dale, 2000). Briefly, this process includes transformation to Talairach space, intensity normalization for correction of magnetic field inhomogeneities, and removal of non-brain tissues (i.e., skull-stripping). Visual inspection of surface models assessed motion artifact, and manual editing of surfaces was applied in cases where there was overinclusion of skull, pial matter, or white matter. Following visual inspection of data, FreeSurfer's white matter segmentation, derived from the Desikan-Killiany atlas (Desikan et al., 2006), defined sub-cortical frontal white matter regions relative to overlying cortex. We applied estimated total intracranial volume (eTIV), generated by FreeSurfer, as a covariate to linear models to account for differences in head size across participants.

#### Region-Specific White Matter Hyperintensities

Using Functional Magnetic Resonance Imaging of the Brain (FMRIB) Software Library v6.0 Linear Image Registration Tool (FSL FLIRT) (Jenkinson and Smith, 2001; Jenkinson et al., 2002; Greve and Fischl, 2009) each participant's native space MPRAGE was coregistered to their FreeSurfer atlas, saving each participant's registration parameters. The registration parameters were applied using FSL FLIRT to linearly transform each participant's LST-derived binary lesion map to their FreeSurfer white matter segmentation. This assigned lesions to their respective Desikan-Killiany white matter regions. Then using FreeSurfer's mris_anatomicalstats, number of voxels for total region volume and amount of WMH load for each FreeSurfer white matter region were calculated. To adjust for individual differences in region size, region-specific WMH load was divided by total region volume. A constant of 1 was added and a log transformation was applied to normalize fractional WMH distribution. We exported right and left hemisphere white matter regions to the Statistical Package for the Social Sciences (SPSS; IBM Corp. Released 2017. IBM SPSS Statistics for Macintosh, Version 25.0. Armonk, NY, U.S.A.) for further analysis.

**Figure 2:**
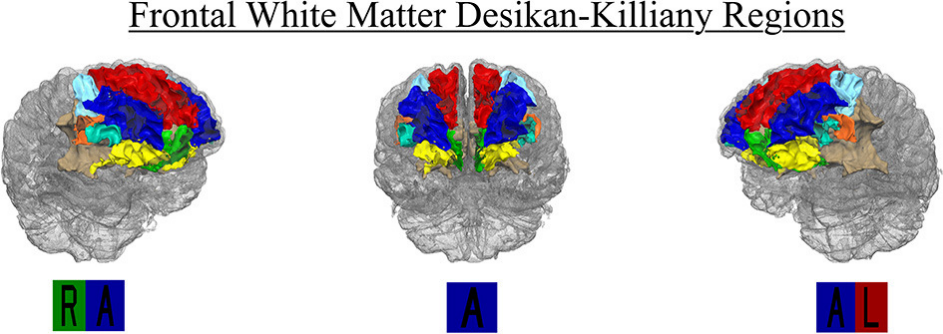
Region-specific WMH Desikan-Killiany ROIs included in final analysis (*n* = 16). Regions included right and left hemisphere Superior Frontal (red), Rostral (blue), and Caudal (light blue) Middle Frontal, Medial (green) and Lateral Orbitofrontal (yellow), Pars Opercularis (orange), Pars Triangularis (cyan), and Periventricular/Deep Frontal (beige).

FreeSurfer provides 35 segmented regions of interest (ROI) in each hemisphere. The FreeSurfer segmentation use the Desikan-Killiany atlas to label white matter regions relative to nearby gray matter. The white matter regions at the heart of our analysis consisted of WM segmentations to a depth 5 mm below each corresponding cortical region. Regions beyond this boundary are labeled “Unsegmented White Matter.” Superficial frontal white matter ROIs (i.e., nearest to the gray matter boundary) include Superior Frontal, Rostral and Caudal Middle Frontal, Pars Opercularis, Pars Triangularis, Pars Orbitalis, Lateral and Medial Orbitofrontal, Precentral, Paracentral and Frontal Pole. There were no participants with any lesioned voxels in the bilateral Frontal Pole, and only 2.4 and 1.7% of participants with any lesioned voxels in the left and right Pars Orbitalis, respectively. Therefore, those two regions were removed from analyses. The Precentral and Paracentral areas are known for sensory and motor integration. We did not have specific hypotheses for these regions related to executive function, and they were therefore excluded from analyses to reduce the number of comparisons. Given past relationships with periventricular WMH and cognition, the authors used in-house processing in MATLAB to restrict the FreeSurfer Unsegmented White Matter region to the frontal lobe. The posterior boundary of the FreeSurfer Superior Frontal region was used to parse the Unsegmented White Matter region anteriorly. The resulting region was labeled as “Periventricular/Deep Frontal,” and volume and lesion load were calculated. Final analyses included eight right and left hemispheres regions for a total of 16 ROIs: Superior Frontal, Rostral and Caudal Middle Frontal, Pars Opercularis, Pars Triangularis, Lateral and Medial Orbitofrontal, and Periventricular/Deep Frontal regions (Figure 2). Mean WMH load for the entire sample is located in Table 3.

**Table 3:** Average frontal WMH load by ROI.

**Frontal WMH ROI**	**Left hemisphere**	**Right hemisphere**
	**Mean (SD)**	**Mean (SD)**
Caudal Middle Frontal	0.002 (0.008)	0.002 (0.006)
Rostral Middle Frontal	0.001 (0.005)	0.001 (0.004)
Lateral Orbitofrontal	0.004 (0.009)	0.002 (0.005)
Medial Orbitofrontal	0.0002 (0.001)	0.0003 (0.001)
Pars Triangularis	0.003 (0.009)	0.002 (0.009)
Pars Opercularis	0.001 (0.005)	0.002 (0.009)
Superior Frontal	0.001 (0.002)	0.001 (0.002)
Periventricular/Deep Frontal	6.210 (1.357)	6.200 (1.240)

*All values were log-transformed*.

### Data Analyses

Analyses were performed in SPSS. Multiple linear regressions measured the unique effect of region-specific lesion volume and NIHTB performance. All models controlled for age, sex, education, multisite scanner differences, eTIV, and CVD risk. High CVD risk (*n* = 136, 48.7%) was defined as self-report of two or more of the following: prior or current smoker status, history of angina, atrial fibrillation, cardiac arrest, coronary artery disease, hypertension, high cholesterol, heart attack, heart failure, stroke, transient ischemic attacks, and diabetes (Bangen et al., 2014). For primary analyses, multiple independent linear regressions investigated the relationship between performance on NIHTB executive function subtest performance (DCCS, Flanker, List Sorting), and region-adjusted WMH load in all available right and left hemisphere frontal ROIs. False Discovery Rate (FDR) correction was applied for each subtest on all 16 ROIs using the Benjamini-Hochberg Method (Haynes, 2013).

## Results

### Frontal WMH Load and NIHTB Executive Function

For each of the three NIHTB executive function tests, we examined the contribution of number of WMH voxels in a frontal ROI (adjusted for region size) individually alongside predictors including age, sex, education, MRI scanner differences, eTIV, and CVD risk. FDR correction adjusted for multiple comparisons across frontal ROIs, while covariates were consistent between models. For all models, FDR-corrected p-values of regional frontal WMH load are located in Table 4.

**Table 4:** Results for left and right hemisphere WMH in NIH toolbox executive functioning.

**FreeSurfer region**	**DCCS**		**Flanker**		**List sorting**	
	**LH FDR *p*-value**	**RH FDR *p*-value**	**LH FDR *p*-value**	**RH FDR *p*-value**	**LH FDR *p*-value**	**RH FDR *p*-value**
Superior Frontal	0.02^*^	0.02^*^	0.06	0.02^*^	0.93	0.93
Medial Orbitofrontal	0.08	0.02^*^	0.64	0.18	0.93	0.93
Caudal Middle Frontal	0.07	0.08	0.48	0.64	0.93	0.93
Rostral Middle Frontal	0.07	0.09	0.65	0.64	0.93	0.93
Lateral Orbitofrontal	0.50	0.08	0.64	0.48	0.93	0.93
Periventricular/Deep Frontal	0.08	0.08	0.48	0.18	0.93	0.93
Pars Opercularis	0.41	0.48	0.65	0.82	0.93	0.93
Pars Triangularis	0.51	0.45	0.64	0.48	0.93	0.93

*All models included age, sex, education, multisite scanner differences, and cardiovascular disease risk. β = standardized beta weight*,

* *FDR p-value significant < 0.05*.

### Dimensional Change Card Sort—Set-Shifting/Switching

**Figure 3:**
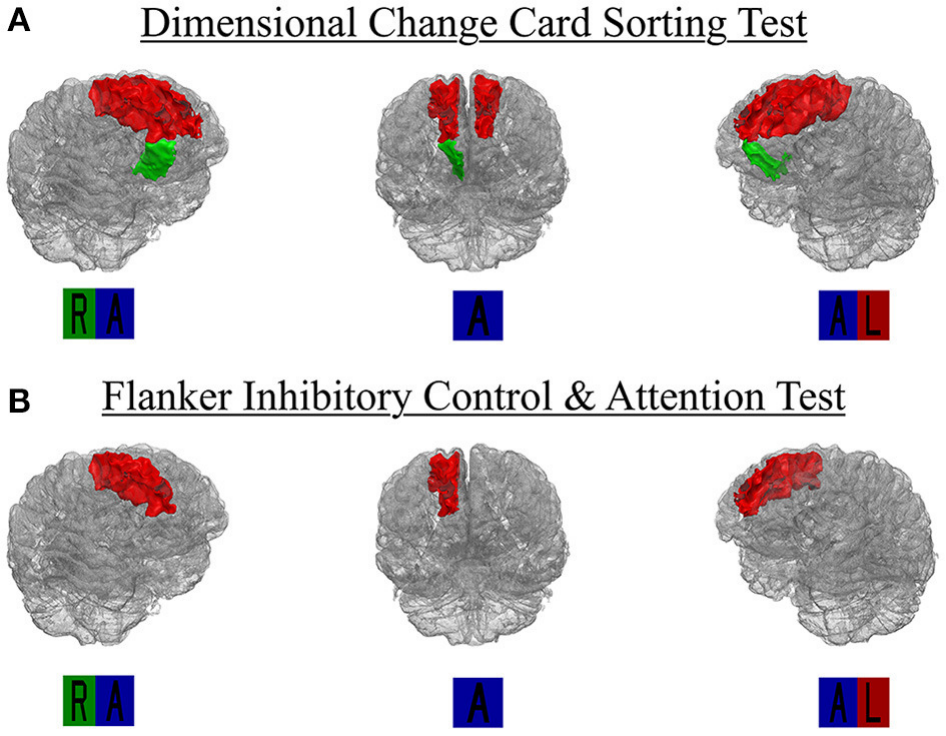
Region-specific WMH ROIs significant for **(A)** dimensional change card sort test and **(B)** Flanker inhibitory control and attention test. Significant Desikan-Killiany regions rendered as 3D isosurface mesh files and presented in surface. R, right; L, left; A, anterior.

**Figure 4:**
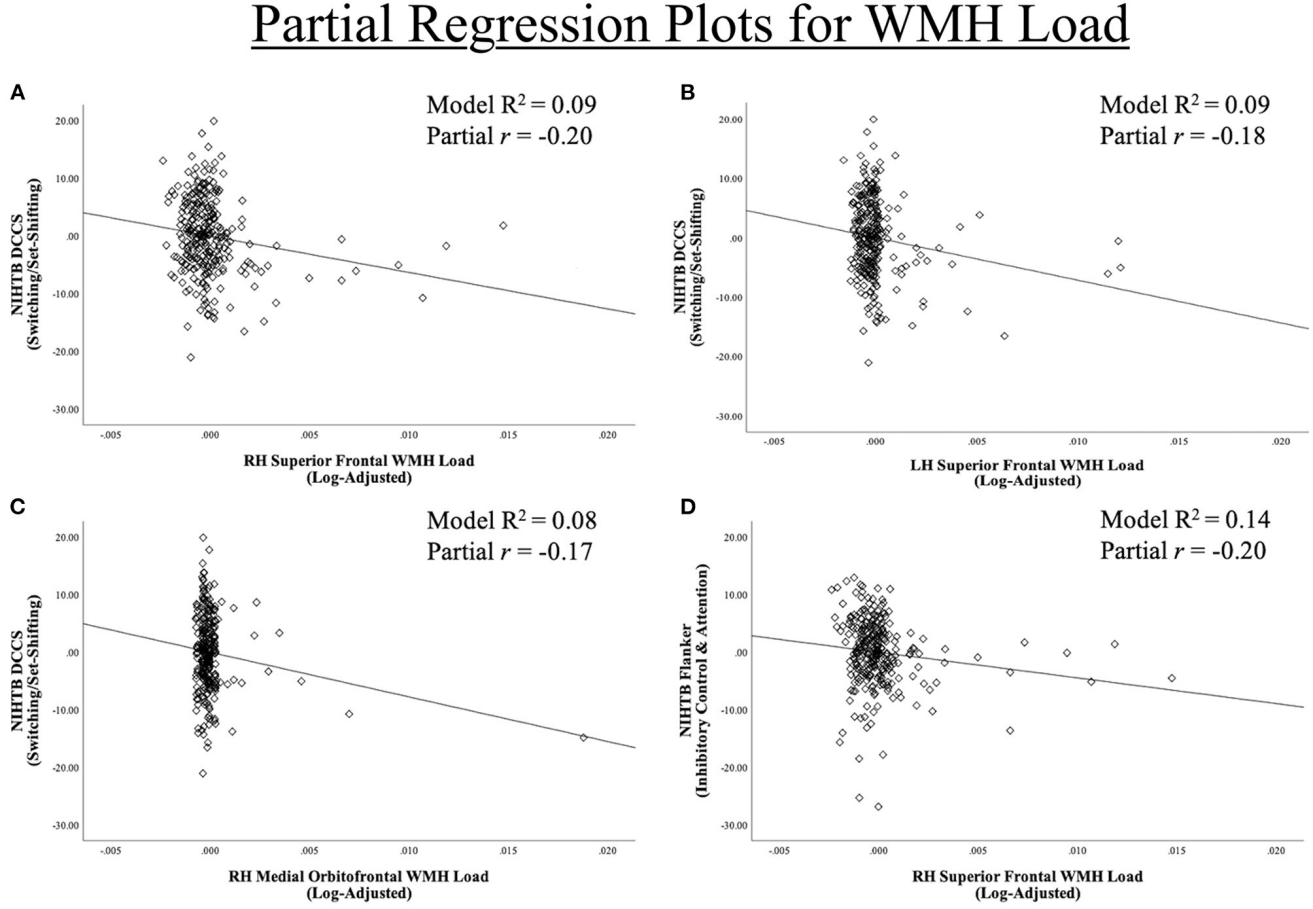
Region-Specific WMH ROIs significant for **(A–C)** dimensional change card sort test and **(D)** Flanker inhibitory control and attention test. Partial regression scatter plots for relationships between NIHTB DCCS and Flanker performance and significant Desikan-Killiany frontal regions WMH load for **(A)** left hemisphere superior frontal, **(B,D)** right hemisphere superior frontal, **(C)** right hemisphere medial orbitofrontal.

Each of the 16 models examining DCCS performance were significant overall, explaining 5.7–9.2% variance in set-shifting score. Poorer DCCS performance associated with higher WMH load in three regions beyond effects of covariate predictors. Following FDR-correction, higher WMH load was associated with poorer DCCS performance in one bilateral ROI, the superior frontal region. Poorer DCCS performance was also unilaterally related to higher WMH load in one ROI, the right medial orbitofrontal region (Figures 3, 4A–C). Regarding covariates, higher education and eTIV were associated with improved DCCS performance in each model. The standardized beta-weights ranged from 0.14 to 0.16 (all *p*-values < 0.03) for education and 0.17–0.19 (all *p*-values < 0.04) for eTIV suggesting a standard unit increase in DCCS performance was associated with roughly 0.15 unit increase in education and roughly 0.18 unit increase in eTIV. In contrast, there were no models where age, sex, multi-site scanner differences, or CVD risk were significantly related to DCCS performance.

### Flanker Inhibitory Control and Attention Test—Inhibition

Each of the 16 models examining Flanker performance were significant overall, explaining 10.2–13.9% variance in inhibition score. Following FDR-correction, poorer Flanker performance associated with higher WMH load in one region beyond effects of covariate predictors, the right superior frontal region (Figures 3, 4D). Regarding covariates, lower age was associated with higher inhibition performance for all models, except the model where WMH load was significant (right superior frontal) with standardized beta-weights ranging from −0.12 to −0.16 (all *p*-values < 0.05). In contrast, higher education was associated with better Flanker performance in each model with standardized beta-weights ranging from 0.14 to 0.16 (all *p*-values < 0.03). Therefore, a standard unit increase in Flanker performance was associated with roughly 0.15 units increase in education. Additionally, there were significant differences between study site in Flanker performance with the UA cohort scoring 2.23 points higher on average compared to the UF cohort (Table 2). Thus, the 16 models were re-run separately for each site. Left and right superior frontal findings remained for UF, but not UA. Additionally, there were no differences between sites in WMH load for any of the 16 regions suggesting differences in NIHTB performance variance, rather than acquisition differences between MRI scanners. Finally, there were no models where sex or CVD risk were significantly related to Flanker performance.

### List Sorting Working Memory Test—Working Memory

Each of the 16 models examining List Sorting performance were significant overall, explaining 10.1–10.7% variance in working memory score. Following FDR-correction, poorer List Sorting performance was not associated with WMH load in any frontal regions beyond effects of covariate predictors. Regarding covariates, lower age was associated with higher working memory performance for all models with standardized beta-weights ranging from −0.17 to −0.20, suggested that each standard unit increase in List Sorting performance was associated with roughly 0.185 units decrease in age (all *p*-values < 0.02). Additionally, higher education associated with higher List Sorting performance in each model. The standardized beta-weights ranged from 0.17 to 0.18 (all *p*-values < 0.01) for education. Finally, higher eTIV associated with higher List Sorting performance in each model, except the right pars triangularis, with standardized beta-weights ranging from 0.15 to 0.16 (all *p*-values < 0.05) for eTIV. These results suggest a standard unit increase in List Sorting performance was associated with roughly 0.155 units higher education and roughly 0.185 units higher eTIV. In contrast, there were no models where sex, multi-site scanner differences, or CVD risk were significantly related to List Sorting performance.

## Discussion

Our aim was to describe the effect of frontal WMH presence on executive function subtest performance. The results supported our hypotheses that WMH in several frontal regions would be related to poorer performance in executive tasks including set-shifting and inhibition. However, we found no relationship with frontal WMH and working memory performance. Results from this cross-sectional analysis of older adults without neurodegenerative disease emerged above and beyond predictors known to be associated with WMH presence, including age and CVD risk, which is highly associated with future risk of stroke or conversion to dementia (Bangen et al., 2014; Prins and Scheltens, 2015; Moroni et al., 2018). Additionally, it is well-established that WMH initially emerge surrounding the ventricles (i.e., periventricular regions) and extend into superficial brain structures as older adults continue to age (Silbert et al., 2008; Moroni et al., 2018; Dadar et al., 2019). In our sample of healthy older adults, we did not find associations in periventricular/deep frontal regions. The results presented here suggest that the spread of macroscopic white matter changes to superficial regions (i.e., those 5 mm below the cortical boundary) may play an influential role in the neuropsychological profile of cognitive aging. Findings from this study reveal that atlas based region-specific WMH are a sensitive metric for obtaining neural correlates of age-related cognitive changes, prior to the onset of MCI, dementia, or neurological brain disease.

Previous studies have found relationships between global and macroscopic white matter changes and specific domains of cognition including executive function, memory, motor speed, and speed of processing across older adult populations (Wakefield et al., 2010; Brickman et al., 2011; Smith et al., 2011; Meier et al., 2012; Birdsill et al., 2014; Hirsiger et al., 2017; Lampe et al., 2017; Dhamoon et al., 2018; Bangen et al., 2019). The present study extends prior findings by examining the unique subcomponents of executive functions using the NIH Toolbox. We examined performance relative to the presence of frontal lobe WMH healthy older adults. Evidence suggests that declines in executive function are some of the most important changes in normal aging, hindering people's ability to quickly and effectively navigate their environment (Murman, 2015). Previous studies demonstrate associations between total lesion volume and periventricular WMH and cognitive decline, likely due to disruption of long-range axonal connections (Söderlund et al., 2006; Silbert et al., 2008; Wakefield et al., 2010; Dhamoon et al., 2018; van Rooden et al., 2018). Notably, frontal periventricular WMH did not predict poorer cognition in our sample. We extend recent findings by demonstrating superficial WMH are strongly associated with executive functioning performance in healthy individuals (Brugulat-Serrat et al., 2020). The findings presented are clinically relevant because they suggest that the spread of WMH into superficial frontal regions associate with age-related declines in critical cognitive processes including set-shifting/switching between stimuli and inhibiting prepotent responses.

The investigation of region-specific WMH is a growing area of research. One challenge of this research is that several white matter bundles exist in a given region, all of which are thought to be part of larger neural networks projecting across the brain. While white matter tracts are not readily defined by MPRAGE or FLAIR, there is a breadth of literature describing functions of the overlying cortical regions that can guide our interpretation of the effect of white matter disruptions in these regions. The regions included in this study are characterized by white matter location relative to overlying cortex, using the Desikan-Killiany atlas. The frontal regions are areas commonly associated with executive function, memory, and processing speed and represent key neural correlates of age-related cognitive decline in older adults. For example, we found that region-specific WMH in three frontal regions (bilateral superior frontal, right medial orbitofrontal) and one frontal region (right superior frontal) were associated with switching/set-shifting and inhibition, respectively. While we found no relationships for working memory within our ROIs, previous research suggests poorer working memory performance may be related to WMH presence in cingulate or parietal regions, which were not examined in this study (Van Petten et al., 2004; Kennedy and Raz, 2009).

We aimed to control for several contributing factors within our regression models. Cooley et al. posited that age-related cognitive changes are typically associated with degradation in anterior regions of the brain (Cooley et al., 2015), which suggests age is an imperative predictor. Education is also highly related to neuropsychological test performance (Lam et al., 2013). The present study also covaried for increased cerebrovascular risk and found no association between increased CVD risk and presence of region-specific WMH across any of the Desikan-Killiany frontal regions. Regardless of etiology, white matter changes to the regions implicated in the present study appear to impact older adults' ability to perform tasks of executive functions. Disruption to the underlying white matter may be informative for understanding physiological processes contributing to age-related cognitive decline. Future studies examining white matter tractography or functional connectivity could examine these regions as network seeds and examine how disruption of communication through underlying networks is related to declines in executive function.

To our knowledge, we are the first study exploring NIHTB executive function subtest performance in the context of WMH and cognitive aging. Additionally, we are aware of only one prior study examining spatial specificity of WMH and cognitive decline in a non-demented sample of older adults (Lampe et al., 2017). Lampe et al. examined region-specific WMH using a voxel-based approach within specific domains of cognition using cognitive factor scores from a battery including the Consortium to Establish a Registry of Alzheimer's Disease (CERADplus), a German vocabulary test (Wortschatztest), and structured interview measures. Our findings were similar to patterns found in the Lampe et al. study, as well as voxel-wise investigations in populations of older adults with MCI or other neurodegenerative diseases. Across studies, the presence of WMH in frontal, temporal, parietal, and occipital regions associated with several domains of fluid cognitive decline in older adults (Carmichael et al., 2012; Birdsill et al., 2014; Lampe et al., 2017; Jiang et al., 2018; Bangen et al., 2019). Regions of WMH burden in non-demented older adults are less widespread but overlap when compared to neurodegenerative disease populations. We hypothesize that spatial patterns of WMH may serve as a key early marker of future functional decline for older adults experiencing normal age-related cognitive decline as well as those with MCI or dementia. Indeed, one previous study found that baseline global WMH and hippocampal volume are associated with conversion from normal cognition to MCI (Bangen et al., 2018). However, they found that annualized change in global WMH was not significantly associated with conversion to MCI. Use of a regional approach may help to elucidate key regions of longitudinal change associated with conversion. For example, using an atlas based approach, one previous study demonstrated that the WMH pattern in older adults increase over time and progress differently between phenotypes of MCI (i.e., amnestic, non-amnestic) (Bangen et al., 2019). Bangen et al. found that increases in occipital WMH are greater in those with non-amnestic MCI compared to amnestic and cognitively normal controls. Therefore, there may be potential diagnostic value to characterizing region-specific WMH patterns prior to the onset of MCI or dementia. Further research is needed to elucidate how WMH in the regions found here are associated with declines in other domains of cognition.

### Strengths and Limitations

Strengths of the study include a large sample of older adults absent of neurodegenerative disease, using an emerging tool for measuring cognition, the NIH Toolbox. The NIHTB uses item response theory to efficiently measure several cognitive domains, as well as executive function subcomponents. The present study used an atlas-based approach to gain more specificity in identifying key frontal regions where presence of WMH is related to executive function decline between subcomponents. Using the atlas-based approach, we examined the spread of WMH in both superficial and periventricular/deep frontal white matter regions. The present study assessed each ROI using multiple independent regressions. Given issues of multicollinearity, we were not able to assess all ROIs simultaneously. An alternative approach would be to examine these WMH using a voxel-by-voxel approach and determine which coordinates survived. Nonetheless, atlas based approaches have been shown to be a sensitive metric across several recent studies (Lampe et al., 2017; Jiang et al., 2018; Bangen et al., 2019). This study used only an MPRAGE and FLAIR image to characterize WMH, which do not allow for the assessment of white matter bundles. It would be valuable to characterize the impact of these lesions on long-range axonal connections using diffusion weighted imaging, for example. Additionally, this study accounted for CVD risk. Increased vascular risk is associated with age-related brain changes, including reduced cerebral blood flow and presence of WMH (Debette et al., 2011; Bangen et al., 2014). However, we know of no studies that have found region-specific differences beyond that of CVD risk. Moreover, prior research demonstrates that the effect of WMH on cognition is mediated by cortical atrophy (Rizvi et al., 2018). The present study accounted for this effect by adjusting region-specific WMH by region volume within each subject.

The findings presented here demonstrate that WMH are a small, but important, factor for assessing age-related cognitive decline. Alongside other relevant predictors, our models explained roughly 8–10% of executive functioning performance. Nonetheless, our study opens questions for future research to explore other relevant factors that explain cognitive changes in aging. While the cross-sectional design cannot capture dynamic longitudinal white matter or vascular change, our study provides a foundation for future large-scale longitudinal studies in older adults and executive function. This is an important next step to pursue to determine the trajectories of older adults with WMH and how they might predict functional outcomes. Additionally, the present study only examined older adults without evidence of major cognitive decline and did not include a dementia group (or other neurodegenerative disease types) for comparison. The original protocol excluded participants with formal diagnosis or evidence of MCI, dementia (i.e., Uniform Data Set *z*-score < −1.5), major psychiatric disease, and those with contraindication to MRI, which resulted in a more homogeneous sample, but a lack of comparison groups. Additionally, our sample was largely Caucasian and, on average, highly educated. Presence of WMH, CVD risk, and several other factors may be differentially represented in non-white and less than college educated participants.

There are a range of possible settings for automated lesion segmentation that can influence lesion identification. The software and lesion identification thresholds used in this study match those of previously published data in older adults (Birdsill et al., 2014). We also carefully examined both of LST's processing pipelines (prediction vs. growth) and found consistent results (See Supplementary Figure 1). Overall, the selection of the automated lesion thresholds in this study performed within expectations. Careful attention to segmentation performance should also occur in future studies seeking to replicate findings across study populations and scanning parameters. Further still, more methodological research on the ideal lesion segmentation settings across samples and scanner would be an asset for furthering the use of LST in various clinical and research settings.

## Conclusion

The present study explored relationships between region-specific frontal WMH presence and executive function subtest performance in older adults without frank neurodegenerative disease. The results suggest that atlas based, region-specific WMH load may be a sensitive proxy of subtle neuroanatomical and age-related cognitive changes. These region-specific changes may be responsible for previously reported findings of relationships between global WMH and cognition in older adults with MCI and dementia. The older adults included in this study displayed widespread WMH and specific regions appear to be more strongly related to executive function decline compared to the frontal lobes in their entirety. Additionally, higher frontal WMH load associated with poorer performance on tasks assessing switching/set-shifting and inhibition, but not working memory. These differences remained even when controlling for CVD risk, which is thought to be one of the leading etiologies of WMH. Location and spread of WMH in these specific regions may be a precursor to future neural and cognitive changes that occur in aging. Given that WMH are presumed to be of vascular origin, there may be modifiable factors that, if identified early, could provide early markers of future potential adverse outcomes and provide a target for early intervention. Future studies should utilize longitudinal designs capable of elucidating whether WMH in otherwise healthy older adults precedes neuropsychological dysfunction that may ultimately impact ability to perform daily activities and maintain functional independence.

## Data Availability Statement

The datasets presented in this article are not readily available because, the data are managed under the data sharing agreement established with NIA and the parent R01 clinical trial Data Safety and Monitoring Board in the context of an ongoing Phase III clinical trial (ACT study, R01AG054077). All trial data will be made publicly available 2 years after completion of the parent clinical trial, per NIA and DSMB agreement. Requests for baseline data can be submitted to the ACT Publication and Presentation (P&P) Committee and will require submission of a data use, authorship, and analytic plan for review by the P&P committee. Requests to access the datasets should be directed to ajwoods@phhp.ufl.edu.

## Ethics Statement

The studies involving human participants were reviewed and approved by The Institutional Review Board (IRB) at the University of Florida and University of Arizona. The patients/participants provided their written informed consent to participate in this study. Written informed consent was obtained from the individual(s) for the publication of any potentially identifiable images or data included in this article.

## Author Contributions

EB, AA, JK, NE, HH, PB, SD, GA, RC, and AW contributed text to the manuscript. EB, AO'S, AA, and AW performed data analysis. SD, GA, and AW contributed to manuscript revisions. All authors provided edits and approved the final version of the manuscript.

## Conflict of Interest

The authors declare that the research was conducted in the absence of any commercial or financial relationships that could be construed as a potential conflict of interest.
