# Supplementary material for: Frontal White Matter Hyperintensities and Executive Functioning Performance in Older Adults
Source: Front Aging Neurosci. 2021 Jun 28;13:672535. doi: 10.3389/fnagi.2021.672535 (PMC8273864; doi:10.3389/fnagi.2021.672535)
Supplement: Supplementary file 1 [file Table_1.DOCX]

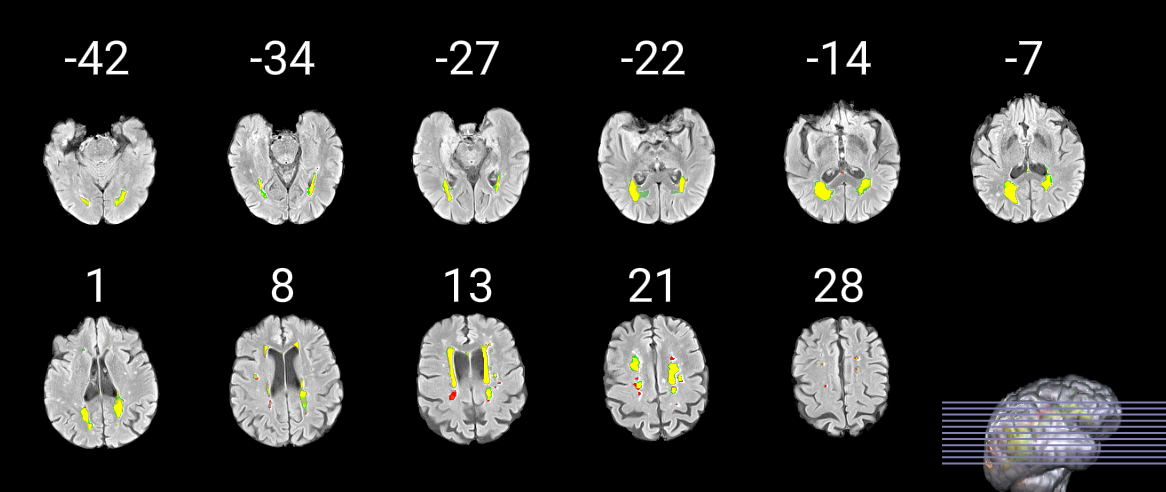


x


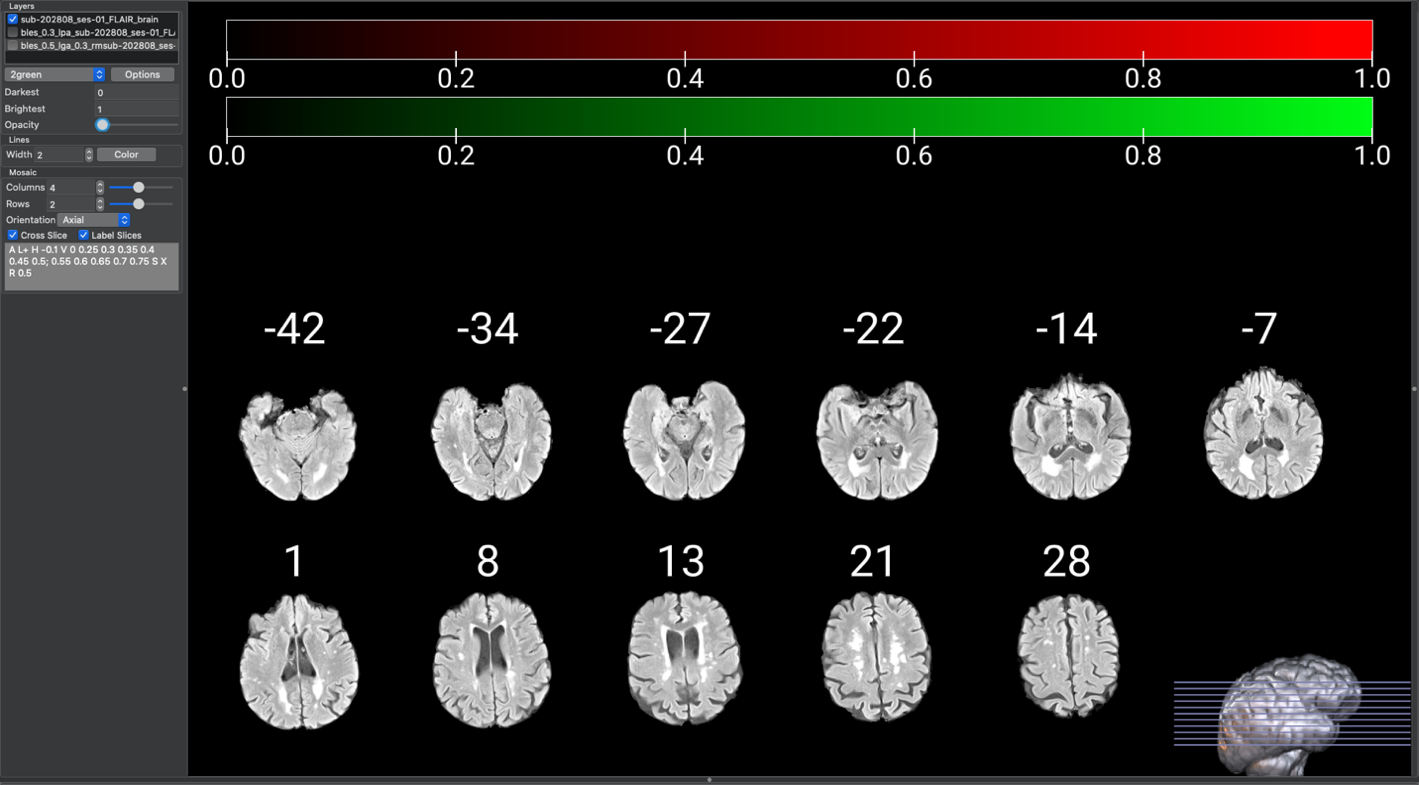

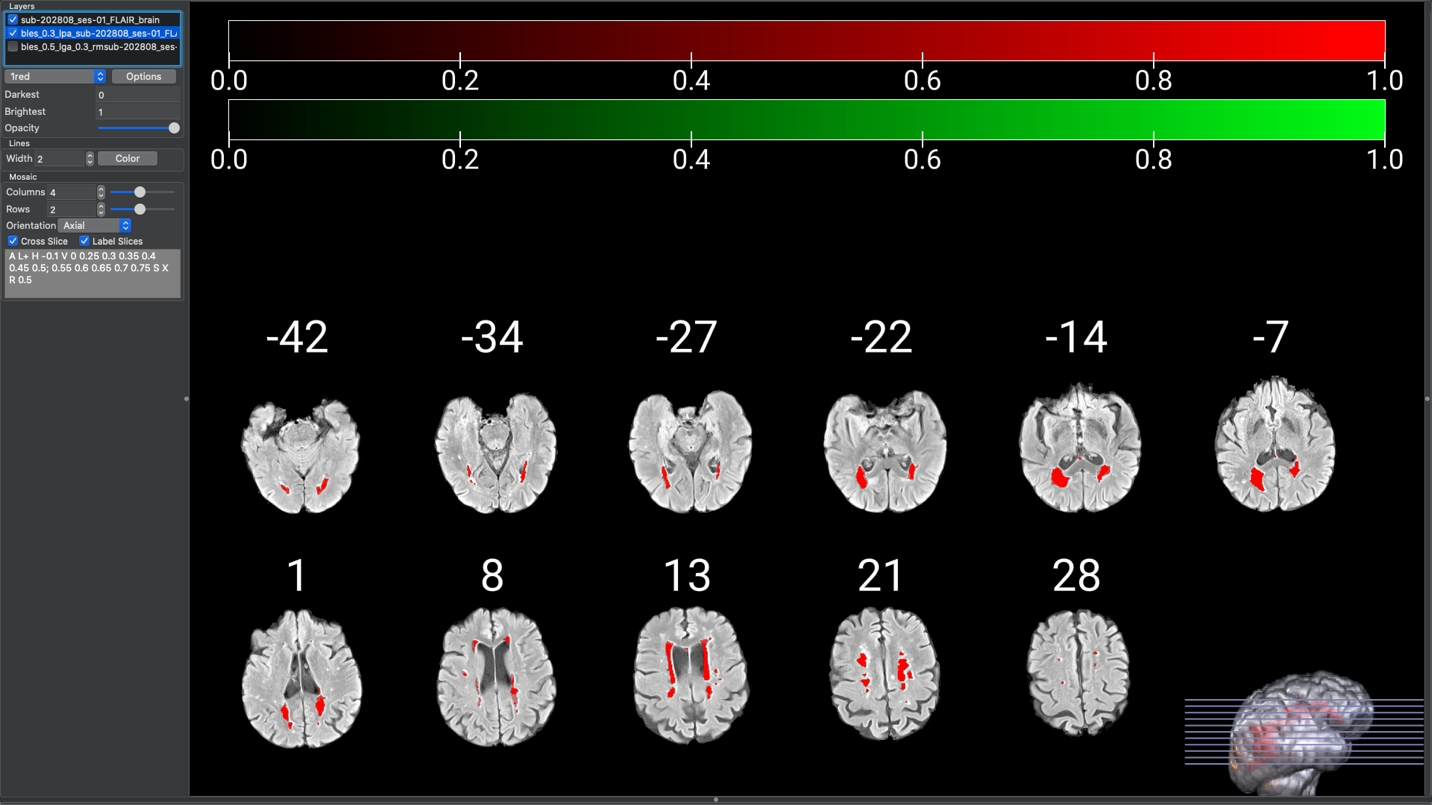


**LESION PREDICTION ALGORITHM, k = 0.3**


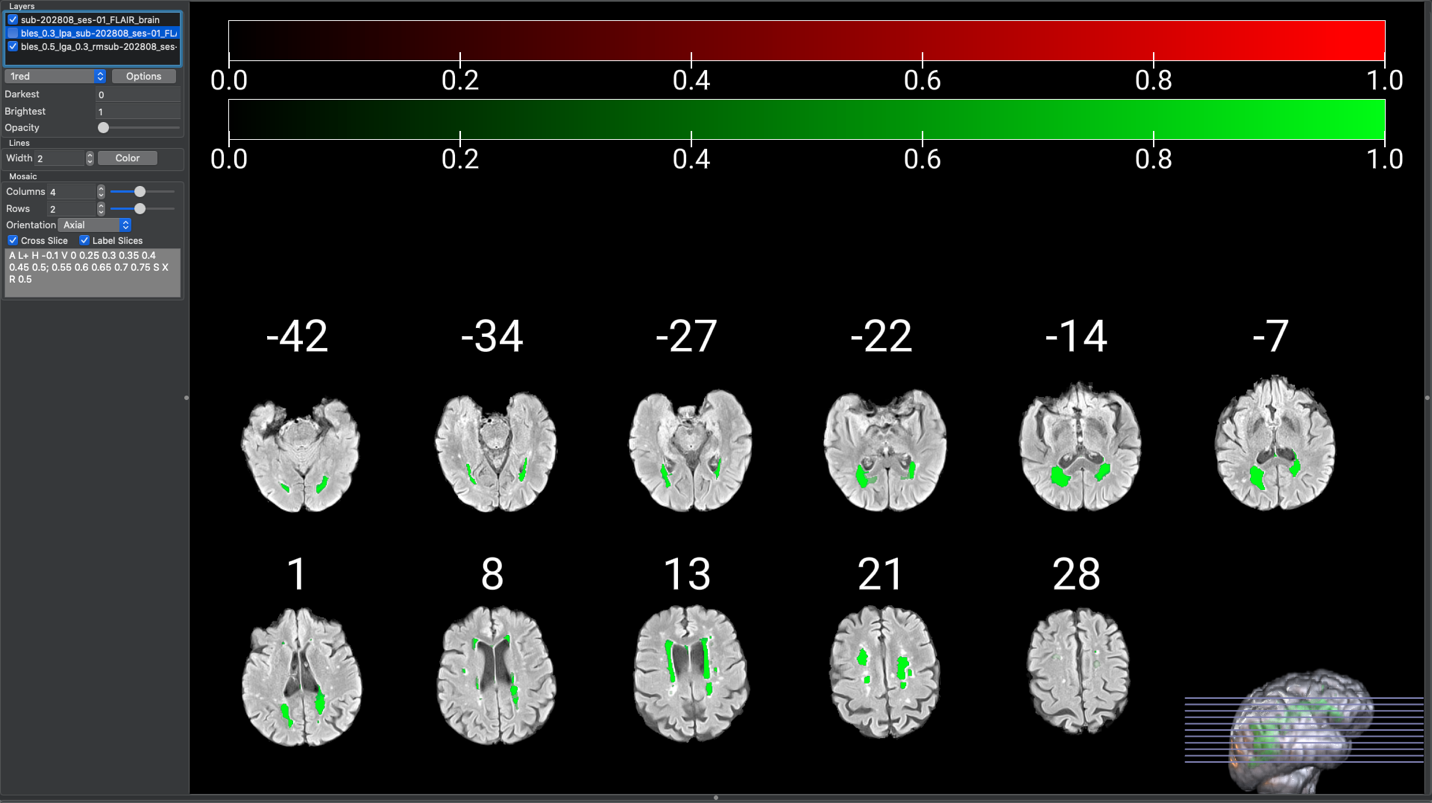


**LESION GROWTH ALGORITHM, k = 0.3**

LESION PREDICTION ALGORITHM

LESION GROWTH ALGORITHM

LESION ALGORITHM OVERLAP

**LESION ALGORITHM OVERLAP**

**Supplemental Figure 1.** Comparison of Lesion Segmentation Tool algorithm, Lesion Prediction Algorithm (LPA) and Lesion Growth Algorithm (LGA).

**REGIONS OF ALGORITHM OVERLAP**
